# Evaluation and Source Apportionment of Potentially Toxic Elements in the Chayuan Reservoir, Guizhou Province Using the Potential Ecological Risk Index (RI) and the PMF Model

**DOI:** 10.3390/toxics14040305

**Published:** 2026-03-31

**Authors:** Xiaolin Feng, Mingfei Zhu, Meimei Yang, Pengfei Wang, Chunchun Chen, Chen Liu, Qiuhua Li

**Affiliations:** 1 Key Laboratory for Information System of Mountainous Area, Protection of Ecological Environment of Guizhou Province, Guizhou Normal University, Guiyang 550001, China; m15180838356@163.com (X.F.); zhumingfei98@163.com (M.Z.); 18083549830@163.com (M.Y.); wangpf_97@163.com (P.W.); 18386252367@163.com (C.C.); 2 Guizhou International Science & Technology Cooperative Base of Aquatic Ecology Research, Guiyang 550001, China; 3 Guizhou Key Laboratory of Advanced Computing, Guiyang 550001, China; 4 School of Cyber Science and Technology, Guizhou Normal University, Guiyang 550001, China

**Keywords:** potentially toxic elements, potential ecological risk index (RI), PMF model, karst reservoir

## Abstract

Understanding the accumulation, ecological risk, and source interactions of potentially toxic elements (PTEs) in reservoir sediments is essential for protecting drinking water safety, yet such processes remain insufficiently understood in karst tea-plantation watersheds influenced by mixed anthropogenic activities. In this study, sediment cores collected from four sites (CY-1 to CY-4) during 2022–2024 were analyzed, and an integrated framework combining the Potential Ecological Risk Index (RI), Spearman correlation analysis, Principal Component Analysis (PCA), and Positive Matrix Factorization (PMF) was applied to evaluate contamination characteristics and quantify source contributions. The results revealed significant spatial–vertical heterogeneity of PTEs, with Zn (up to 153 mg/kg) and Cr (up to 64.6 mg/kg) showing the greatest variability, and strong co-enrichment among Cu, Zn, Pb, and Ni (r > 0.85, *p* < 0.01). Although the overall ecological risk was low (RI = 83.15–106.69), As contributed the highest proportion of risk (28–35%). PCA indicated distinct grouping patterns among elements, while PMF resolved three major sources: domestic sewage and agricultural runoff, agricultural and coal-combustion inputs, and industrial–traffic emissions. Notably, physicochemical parameters (TP, TN, and COD) played important roles in regulating the mobility and partitioning of PTEs by influencing nutrient-associated adsorption processes, organic matter complexation, and redox-related transformations. These findings highlight the multi-source-driven accumulation mechanisms of PTEs in karst reservoirs and provide a scientific basis for targeted pollution control and watershed management in agriculturally impacted regions.

## 1. Introduction

With the accelerating pace of global industrialization and urbanization, potentially toxic elements have become one of the central issues of concern in the field of global environmental ecology [1]. Due to their high toxicity, persistence, and non-degradability [2], potentially toxic elements can remain in environmental media for extended periods and undergo biomagnification through the food chain [3,4], posing serious threats to ecosystem structure and function as well as to human health. In recent years, numerous studies have demonstrated that sediments serve as both sinks and secondary sources of PTEs in aquatic systems [5,6], and their contamination levels directly reflect the changing trends in watershed environmental quality [7,8]. Therefore, systematically assessing the pollution characteristics, ecological risks, and source apportionment of PTEs in reservoir sediments has become an important focus in contemporary environmental science research [9,10]. Previous studies have systematically summarized the occurrence characteristics and source categories of potentially toxic elements (PTEs) in urban-affected environments. These studies indicate that PTEs such as As, Pb, Zn, Cu, and Hg are commonly derived from multiple anthropogenic sources, including industrial emissions, traffic activities, fossil fuel combustion, and agricultural inputs, often exhibiting complex spatial heterogeneity and mixed-source signatures. Such findings highlight the necessity of applying integrated approaches for accurate source apportionment and risk assessment [11].

Guizhou Province, located in southwestern China, is an important ecological function zone and one of the country’s major tea-producing regions [12]. The area’s unique karst geomorphology renders its ecosystems highly sensitive to exogenous pollution [13]. Meanwhile, the combined impacts of mining, smelting, transportation, and agricultural activities have led to a continuous increase in heavy metal concentrations in certain water bodies and sediments [14]. The Chayuan Reservoir in Guizhou not only serves as an irrigation water source for surrounding tea plantations but also plays a vital role in regional ecosystem functions such as climate regulation and hydrological balance [15,16]. However, excessive accumulation of PTEs in reservoir sediments may lead to their remobilization or incorporation into the food chain through resuspension and biological uptake, posing potential risks to tea quality and ecological safety [17]. Owing to the dominance of tea plantations in its watershed, the tea-plantation reservoir is strongly influenced by the application of fertilizers and pesticides, causing metals such as As and Cu to enter the water through runoff [18]. Compared with ordinary reservoirs, its sediment PTEs are more affected by agricultural non-point sources, with enrichment and migration characteristics closely related to soil erosion and particle composition [19]. Therefore, investigating the pollution levels, ecological risks, and sources of PTEs in the sediments of the Chayuan Reservoir is of great scientific importance and provides valuable guidance for regional environmental management and the sustainable development of agriculture [20].

Various methods have been widely applied to assess PTE contamination and sources [21]. including the Potential Ecological Risk Index (RI) [22], Principal Component Analysis (PCA) [23], and receptor models such as Positive Matrix Factorization (PMF) [24]. However, each method has inherent limitations [25], RI focuses on ecological risk but does not provide source information [26]; PCA reveals covariance structures but cannot directly identify pollution sources [27]; and PMF, while capable of quantitative source apportionment, requires supporting evidence for reliable interpretation [28]. Therefore, integrating multiple methods is necessary to achieve a more comprehensive understanding of PTE behavior and sources [29].

Among the available tracing approaches, the Positive Matrix Factorization (PMF) model demonstrates distinct advantages in quantitatively resolving complex multi-source contamination systems [30]. By minimizing the weighted sum of squared residuals, PMF performs a non-negative factorization of the observation matrix, allowing for simultaneous identification of potential pollution sources and quantification of their relative contributions [31]. Compared with traditional methods such as Principal Component Analysis (PCA) and Multiple Linear Regression (APCS-MLR), PMF explicitly accounts for data uncertainties and avoids errors caused by arbitrary rotations or negative constraints, thereby ensuring higher statistical stability and reliability. The resulting factor profiles and contributions provide a robust quantitative basis for revealing the migration and transformation mechanisms of PTEs and for developing targeted management strategies. In this study, multiple statistical approaches were combined to improve the robustness of source apportionment. Correlation analysis and PCA were used as exploratory tools to reveal relationships and grouping patterns among PTEs, while the PMF model was applied as a receptor model to quantitatively identify pollution sources and estimate their contributions. This combined framework allows for more reliable interpretation compared to using a single method.

## 2. Materials and Methods

### 2.1. Study Area

**Figure 1:**
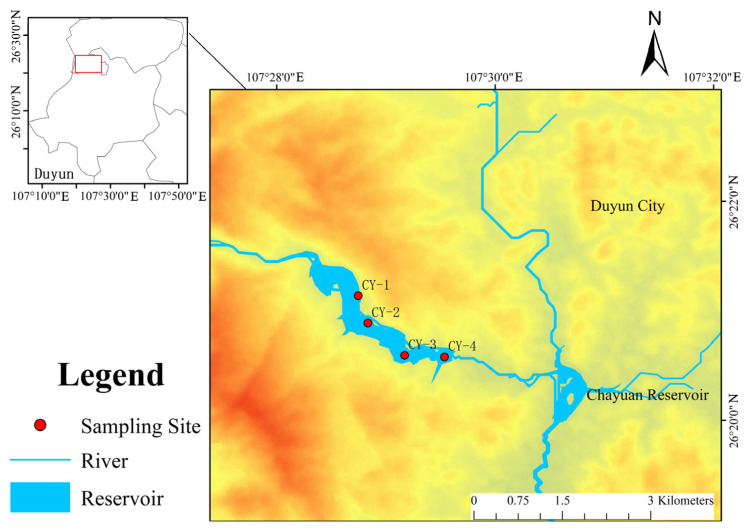
Location map of the study area.

The Chayuan Reservoir is located in Lujiazhai, in the northern suburbs of Duyun City, Qiannan Prefecture, Guizhou Province. It lies in the lower reaches of the Gujiang River, a tributary of the Mawei River, approximately 17 km upstream from downtown Duyun. The reservoir’s catchment area covers 142.3 km^2^, and the river length above the dam site is 25.1 km (Figure 1). The normal storage level of the reservoir is 844.30 m, with a check flood level of 855.80 m, and a total storage capacity of 19.6 million m^3^. The reservoir plays a key role in Duyun’s urban flood control system and serves as the city’s main source of municipal water supply.

Field surveys indicate that the upstream catchment is dominated by forest cover (>60%), with limited industrial development [32]. The study area lies within a subtropical humid monsoon climate zone, where natural environmental conditions are favorable for hydrological regulation and ecosystem stability. Land use in the basin is diverse: forest land accounts for approximately 60% of the total area and plays a key role in water conservation and soil retention; cropland occupies about 25%, primarily used for cultivating rice and maize; urban and construction land accounts for 8%; and water bodies and other land types comprise the remaining 7% (Figure 1).

### 2.2. Collection, Storage, and Pre-Treatment of Sediment Samples

Sediment sampling was conducted in November 2022, April 2023, January 2024, and April 2024. Four sampling sites (CY-1, CY-2, CY-3, and CY-4) were established in the Chayuan Reservoir, where gravity corers were used to collect four sediment cores, each 8 cm in length, representing the recent depositional layer (approximately <5 years based on typical sedimentation rates in subtropical reservoirs) [33]. After collection [34], the cores representing recent accumulation layers were sectioned at 1 cm intervals. The surface sediments were then freeze-dried, and debris such as twigs and stones was removed [35]. The samples were subsequently ground and passed through a 100-mesh sieve to ensure homogeneity and minimize analytical errors [36]. In this study, the concentrations of Hg, Pb, Cr, Cu, Zn, As, and Ni in the sediments were determined using inductively coupled plasma mass spectrometry (ICP–MS) to assess the distribution characteristics and pollution levels of PTEs at different sampling sites. The digestion program included a gradual temperature increase to 180–200 °C over 20–30 min and was maintained for an additional 20 min to ensure complete digestion. This method offers high sensitivity and accuracy and is widely applied in environmental geochemistry and sediment contamination studies [37]. The background values of PTEs adopt the sediment background values in the karst areas of Guizhou Province in “Background Values of Soil Elements in China” [38]. Quality assurance and quality control (QA/QC) procedures were strictly implemented throughout the analytical process. Calibration curves were established using multi-element standard solutions, with correlation coefficients (R^2^) exceeding 0.999 for all elements. Procedural blanks and duplicate samples were included in each batch to monitor contamination and analytical precision.

Certified reference materials (CRMs) were analyzed alongside the samples to verify accuracy, and the recoveries of target elements ranged from 90% to 110%. The relative standard deviations (RSDs) of replicate analyses were generally below 5%, indicating good analytical precision. Method detection limits (MDLs) for all elements were low and suitable for trace-level determination. Replicate samples were averaged before statistical analysis, resulting in *n* = 60.

### 2.3. Data Processing

Data pre-processing was conducted using Excel 2010. Statistical analyses of sediment PTEs and physicochemical properties were performed with SPSS 22.0. Graphs were generated using Origin 8.0, and spatial distribution maps were created with ArcGIS 10.2. Source apportionment of PTEs was carried out using the EPA PMF 5.0 model.

#### 2.3.1. Potential Ecological Risk Index (RI) Method

The Potential Ecological Risk Index (RI) method was proposed by the Swedish scholar Hakanson in the 1980s to quantitatively evaluate the potential ecological hazards posed by potentially toxic elements [26]. This approach effectively reflects the overall risk of multiple PTEs by integrating the elements’ toxicity coefficients, environmental responses, and background concentrations [39]. In recent years, the RI method has been widely applied in ecological risk assessments of sediments in lakes, reservoirs, and estuaries, and has been validated across different regions to evaluate heavy metal contamination levels in lacustrine sediments [40]. The calculation formulas are as follows:

(1) $$RI=\sum_{i=1}^{n}E_{r}^{i}=\sum T_{r}^{i}\times C_{f}^{i}=\sum_{i=1}^{n}T_{r}^{i}\left(\frac{C_{i}}{C_{n}^{i}}\right)$$

Here, RI represents the potential ecological risk index; $E_{r}^{i}$ is the potential ecological risk factor for a single heavy metal element ***i***; $T_{r}^{i}$ is the toxic response factor of the heavy metal element ***i***; $C_{i}$ is the measured concentration of a pollutant in the sediment; and $C_{n}^{i}$ is the corresponding background concentration of that pollutant in the sediment. The toxic response factors for PTEs (Hg Ni Pb, Cr, Cu, Zn, and As) are 40,5, 5, 2, 5, 1, and 10 [41], respectively. According to Hakanson (1980) [26], the ecological risk factor ($E_{r}^{i}$) can be classified into five levels: low risk ($E_{r}^{i}$ < 40), moderate risk (40 ≤ $E_{r}^{i}$ < 80), considerable risk (80 ≤ $E_{r}^{i}$ < 160), high risk (160 ≤ $E_{r}^{i}$ < 320), and very high risk ($E_{r}^{i}$ ≥ 320). An RI value of less than 150 indicates a slight ecological risk; 150 ≤ RI < 300 corresponds to a moderate ecological risk; 300 ≤ RI < 600 represents a considerable ecological risk; and RI ≥ 600 indicates a very high ecological risk. This method demonstrates strong robustness for ecological zoning assessments and pollution level classification [42].

#### 2.3.2. Positive Matrix Factorization (PMF) Model

Unlike traditional factor analysis methods, the PMF model imposes non-negativity constraints on factor scores [43], enabling the differentiation of distinct sources within a mixture and providing greater physical interpretability [44]. Proposed by Paatero and Tapper, PMF is a receptor model with non-negativity constraints that has been widely applied for quantitative source apportionment of pollutants in environmental media. The model decomposes the observed data matrix into a source profile matrix and a source contribution matrix by minimizing the weighted sum of squared residuals, thereby estimating the contribution of each potential pollution source. The basic mathematical formulation is defined as Equation (2):

(2) $$X_{ij}=\sum_{k=1}^{p}g_{ik}f_{kj}+e_{ij}$$

where $X_{ij}$ is the concentration of the ***j***_th_ sediment quality indicator in the ***i***_th_ sediment sample, ***i*** = 1,2…***n***, ***j*** = 1,2…***m***; $g_{ik}$ denotes the contribution of the ***i*** contribution of the kth source in the sediment sample, ***k*** = 1,2…***p***; $f_{kj}$ denotes the concentration of the ***j***_th_ sediment quality indicator in source ***k***; and ***e***_ij_ denotes the residual of the ***j***th sediment quality indicator in the ***i***_th_ sediment sample.

The PMF model decomposes the original concentration data into optimal matrices through iterative calculations. These matrices are determined by minimizing the objective function ***Q***, which is mathematically expressed by Equations (3) and (4):

(3) $$Q_{\mathrm{ture}}=\sum_{i=1}^{n}\sum_{j=1}^{m}{\left(\frac{e_{ij}}{u_{ij}}\right)}^{2}$$

(4) $$Q_{\mathrm{robust}}=\sum_{i=1}^{n}\sum_{j=1}^{m}\left(\frac{e_{ij}}{{h_{ij}u}_{ij}}\right)$$

When $\left|\frac{e_{ij}}{u_{ij}}\right|\leq \alpha ,\;h_{ij}=1$; when $\left|\frac{e_{ij}}{u_{ij}}\right|\geq \alpha ,\;h_{ij}=\left|\frac{e_{ij}}{u_{ij}}\right|/\alpha$, where $u_{ij}$ represents the uncertainty of the *j*th sediment quality parameter in the *i*th sample. The study showed a value of 4 for α. The PMF model provides two ***Q*** values, $Q_{\mathrm{ture}}$ and $Q_{\mathrm{robust}}$. The difference between them lies in that $Q_{\mathrm{ture}}$ is calculated based on all samples, whereas $Q_{\mathrm{robust}}$ is computed after excluding outlier points. If $Q_{\mathrm{robust}}$ equals $Q_{\mathrm{ture}}$, the model lacks robustness. In this study, by manually adjusting the number of factors and the number of iterations, the ratio $Q_{\mathrm{robust}}$/$Q_{\mathrm{ture}}$ tended to converge, with residuals falling between −3 and 3. Under these conditions, the results obtained from running the model are considered relatively reliable.

The PMF model also requires an input file specifying the uncertainties of the sediment quality measurements, as defined by the following equations (Equations (5) and (6), applicable when ***c*** ≥ MDL,

(5) $$u_{ij}=\frac{5}{6}\times \mathrm{MDL}$$

When ***c*** < MDL

(6) $$u_{ij}=\sqrt{{\left(\sigma \times c\right)}^{2}+{(0.5\times \mathrm{MDL})}^{2}}$$

In the equations, ***c*** represents the concentration of the sediment quality parameter, MDL is the method detection limit, and σ is the error fraction. Equations (5) and (6) indicate that the uncertainty consists of two components: the uncertainty from the sediment quality measurement and the uncertainty from the detection limit. According to relevant literature, the error fraction is 0.25. In addition, the PMF model provides the signal-to-noise ratio (S/N), which reflects the reliability of the data. Data with S/N = 2.0 should be manually classified as “strong” reliability, S/N values between 2.0 and 0.2 as “weak,” and S/N < 0.2 as “poor” [45].

The optimal number of factors was determined through an iterative process evaluating (1) the stability of factor profiles across multiple runs, (2) the distribution of scaled residuals (within ±3 for > 95% of data points), (3) the ratio of $Q_{\mathrm{robust}}/Q_{\mathrm{ture}}$ approaching 1, and (4) the physical interpretability of each factor in the context of local land use and pollution sources. A three-factor solution was selected as it yielded chemically coherent source profiles with minimal cross-loading and aligned with known anthropogenic activities in the watershed. Bootstrap (BS) and displacement (DISP) analyses were performed to evaluate model stability.

#### 2.3.3. Principal Component Analysis (PCA)

Principal Component Analysis (PCA) is a type of factor analysis that plays an important role in identifying and interpreting the sources of potentially toxic elements in aquatic sediments [46]. This method reduces the dimensionality of multivariate heavy metal data by integrating correlated variables into several principal components, thereby reflecting the underlying correlation structure and potential source patterns among elements [47]. The eigenvalues and variance contribution rates quantify the extent to which each principal component explains the total variance, revealing the dominant pollution source types and their relative influence [48].

The core idea of PCA is to extract composite factors containing the main information through linear combinations, thereby achieving data simplification and pollution source identification [49]. In practice, researchers often combine PCA with correlation analysis to determine the relationships among elements; high correlation coefficients typically indicate that these elements share common sources or exhibit co-enrichment characteristics [50]. Based on this, further PCA can more accurately identify the contributions of different pollution sources, providing a reliable basis for source apportionment studies of PTEs in sediments [51]. It should be noted that PCA is an exploratory statistical tool that does not directly identify pollution sources but provides supportive information for source interpretation when combined with other methods such as correlation analysis and PMF.

#### 2.3.4. Spearman Correlation Analysis

Spearman rank correlation analysis was applied to evaluate the relationships among potentially toxic elements (PTEs) and physicochemical parameters. This non-parametric method is suitable for identifying monotonic relationships between variables without requiring normal distribution assumptions. The correlation coefficients (r) and significance levels (*p*) were calculated using SPSS 22.0.

## 3. Results

### 3.1. Distribution Characteristics of PTEs in Sediments

**Figure 2:**
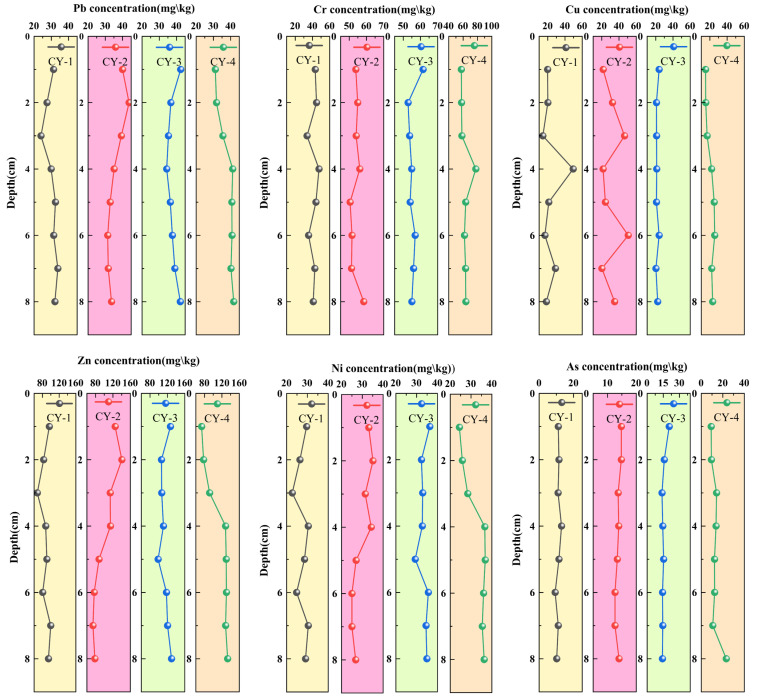
Vertical distribution of PTES (mg/kg).

Figure 2 shows that PTEs in sediments exhibit distinct vertical distribution patterns across different sampling sites. At CY-2, the concentrations of As, Cr, Ni, and other elements fluctuate minimally with depth, showing a relatively uniform distribution; Cu and Pb tend to increase with depth, while Zn and Ni generally decrease. At CY-1, Cu, Zn, and Ni gradually decrease with depth; As and Cr fluctuate little with depth, whereas Pb shows a decrease followed by an increase. At CY-3, Pb, Zn, and Ni generally increase with depth, As and Cr gradually decrease, and Cu exhibits minor fluctuations. At CY-4, the concentrations of As, Cu, Pb, Zn, Cr, and Ni generally increase with increasing depth. The same element also shows differences among sampling sites; for example, As at CY-4 fluctuates with depth, whereas Cu at CY-1 exhibits a decreasing trend.

As shown in Table 1, the statistical results of the concentrations for each type of PTES are presented. Among the sampling sites, the concentrations of Zn, As, and Cr show significant differences, while Cu and Ni exhibit relatively smaller variations. Pb and Cr display a wider concentration range, with several outliers observed. These results indicate that PTEs in the sediments have complex spatial distribution patterns and diverse vertical variation profiles. To evaluate the environmental significance of the measured concentrations, the results were compared with established sediment quality guidelines. Most elements were below their corresponding Threshold Effect Levels (TELs), indicating a low probability of adverse biological effects. However, Zn and As at certain sites approached or slightly exceeded TEL values, suggesting potential ecological concern in localized areas.

### 3.2. Pollution, Toxicity, and Ecological Risk Assessment of PTEs in Sediments

**Figure 3:**
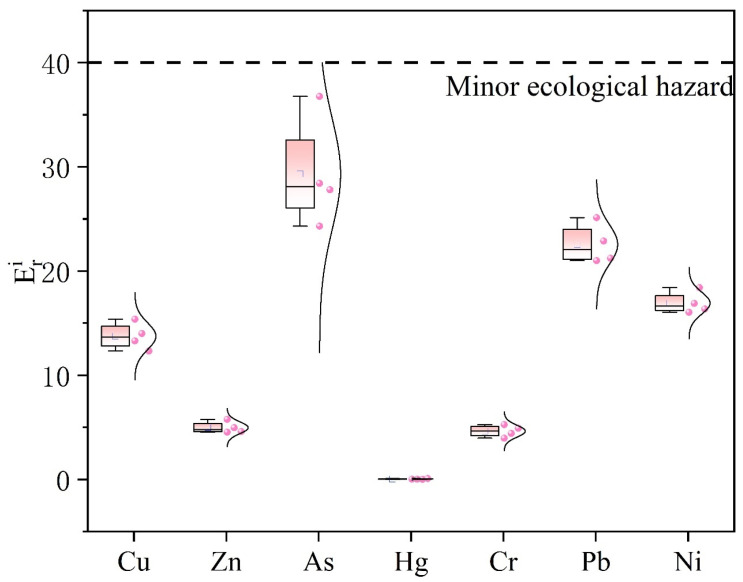
Box plot of potential ecological risk factors ($E_{r}^{i}$) of PTEs.

Table 2 presents the potential ecological risk index for each type of PTEs. The ecological risk index (RI) calculated for the four sampling sites indicates that the overall ecological hazard of the studied metals is low. According to the standards of the Potential Ecological Risk Index, RI values below 150 indicate a low ecological risk. The boxplot in Figure 3 shows that the risk values of As exhibit greater variability, with a median higher than those of other metals, suggesting that As poses a relatively higher risk among all studied metals. In contrast, the risk values of Cu and Zn remain at relatively low levels. Cr and Pb are classified as secondary risk elements. The ecological risk indices of Ni and Cu are low, with relatively narrow ranges. Overall, the sediments in the study area exhibit low risk levels for Cu, Zn, As, Cr, Pb, and Ni, with As showing the most pronounced risk differences among the sampling sites.

### 3.3. Correlation Analysis of PTEs in Sediments

**Figure 4:**
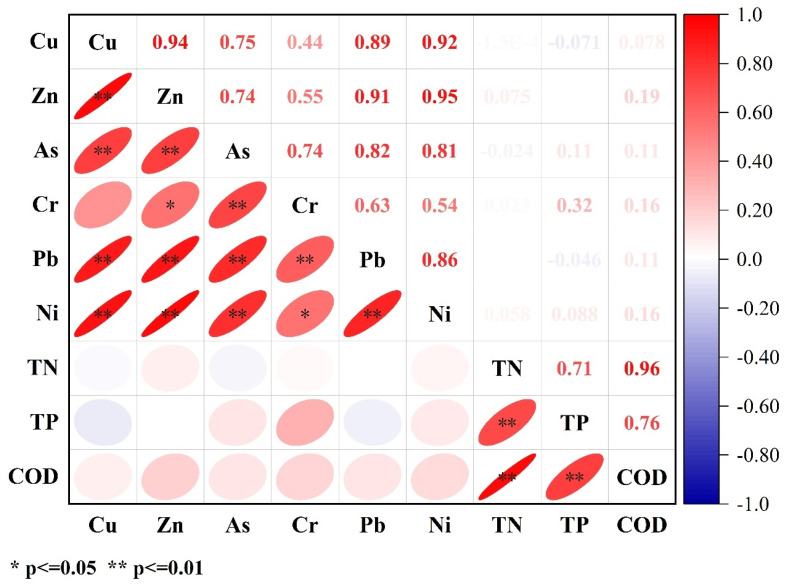
Spearman correlation matrix between PTEs and between PTEs and physicochemical parameters (*n* = 60).

The Spearman correlation analysis provides preliminary evidence of co-enrichment patterns, which is further supported by PCA results. Figure 4 indicates that most PTEs are significantly positively correlated, suggesting similar sources or co-enrichment characteristics. Among them, Cu, Zn, Pb, and Ni exhibit the strongest correlations (r > 0.85, *p* < 0.01), likely reflecting common industrial or anthropogenic sources. As and Cr also show significant correlations with each other but weak correlations with Cu, indicating differences in their geochemical behavior. Hg exhibits the lowest correlation with other PTEs, indicating that its sources or migration mechanisms are relatively independent. Regarding physicochemical parameters, TN shows generally weak correlations with PTEs; TP is weakly positively correlated with As and Pb; and COD displays moderate correlations with Cr, Pb, and Ni, suggesting that organic matter may promote the adsorption or complexation of certain metals. Overall, the distribution of PTEs is primarily controlled by exogenous inputs, with physicochemical factors playing a secondary regulatory role.

### 3.4. Principal Component Analysis (PCA)

**Figure 5:**
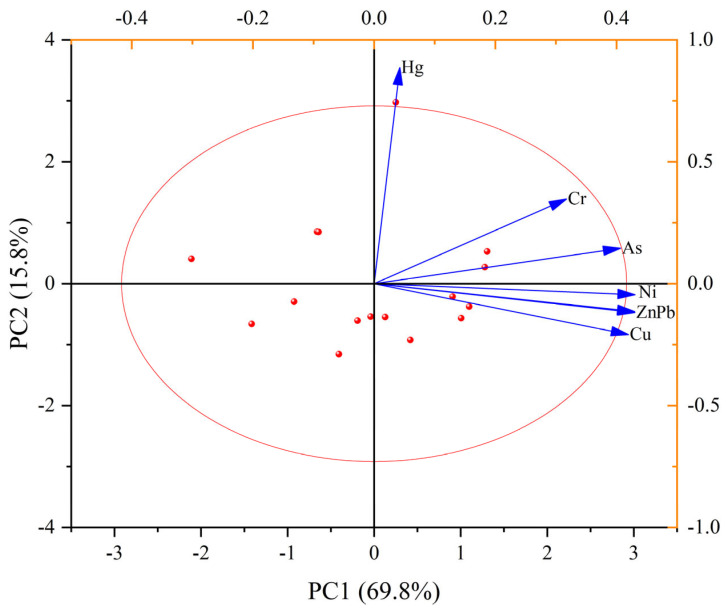
PCA of PTEs in sediments.

Figure 5 clearly reveals the source distribution and covariation relationships of multiple PTEs in the sediments of the study area. The first principal component (PC1) and the second principal component (PC2) together explain 85.6% of the total variance, indicating that the model effectively captures the information in the dataset.

The analysis indicates that the PTEs can be clearly grouped into two primary source clusters. The first group—comprising Cu, Pb, Zn, and Cr—is closely clustered on the positive axis of PC1, exhibiting strong positive correlations. This clustering suggests that these elements share similar variation patterns and may be influenced by common sources, such as emissions from metallurgy, electroplating, or manufacturing activities. The second group, consisting of As and Hg, is distributed along the positive axis of PC2, separated from the first group, indicating independent sources potentially linked to historical pollution from fossil fuel combustion, pesticide application, or specific atmospheric deposition. Notably, Ni is positioned between the two groups, implying that its sources may be a mixture of the aforementioned industrial activities or influenced by other regional factors.

In summary, the PCA results indicate that potentially toxic elements in the study area may be influenced by two distinct anthropogenic activities, providing critical scientific evidence for subsequent precise source apportionment and the development of targeted remediation strategies. Eigenvalues greater than 1 were used as the criterion for component extraction.

### 3.5. Single-Factor Contributions from PMF

**Figure 6:**
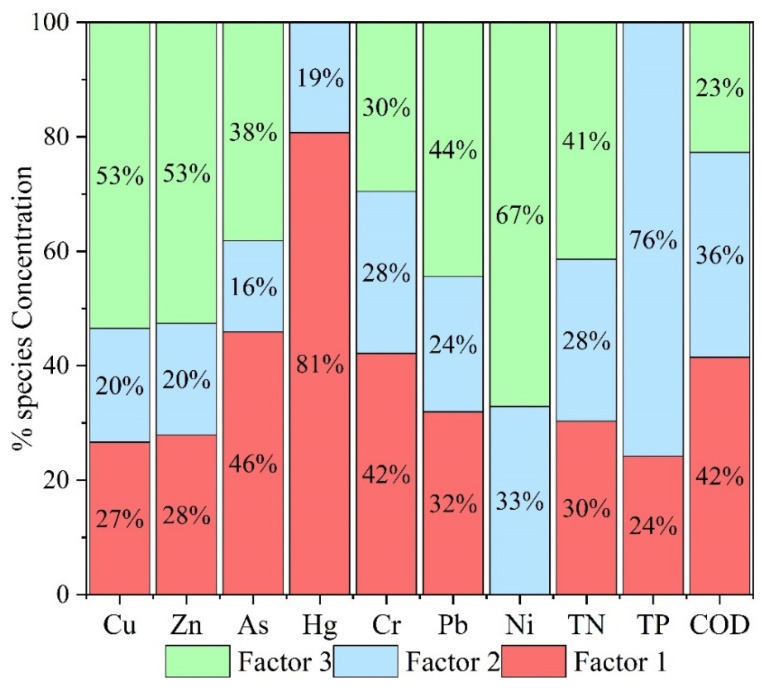
Estimation of the anthropogenic contribution of individual metals in sediment PTEs through the PMF receptor model.

To clarify the sources of PTEs in the sediments, a comprehensive assessment was conducted by integrating correlation analysis, PCA, and the PMF model. The results show that Cu, Zn, Pb, and Ni are highly correlated, indicating that they primarily may be influenced by industrial and traffic activities. As and Cr are significantly correlated but differ notably from the former group, while Hg behaves independently, suggesting distinct sources. PCA results show that Cu, Zn, Pb, and Cr exhibit strong loadings on the same principal component, suggesting that they may be influenced by similar anthropogenic activities, such as industrial emissions, As and Hg reflect coal combustion and agricultural sources, and Ni occupies an intermediate position. The PMF results reveal three potential sources (Figure 6): Factor 1, characterized by COD and Pb, represents domestic sewage and agricultural non-point sources; Factor 2, dominated by Hg, As, Cr, and TP, originates from coal combustion and the use of pesticides and fertilizers; Factor 3, primarily controlling Cu, Zn, Ni, Pb, and TN, reflects industrial emissions and traffic wear. While PCA suggested two important source groups, the PMF model resolved a third factor associated with domestic sewage and organic matter (Factor 1: high loadings of COD and Pb). This discrepancy arises because PCA is an exploratory technique sensitive to covariance structure but unable to quantify source contributions or handle weak signals, whereas PMF, as a receptor model, explicitly accounts for measurement uncertainty and can separate mixed sources with overlapping chemical signatures, such as agricultural runoff and domestic effluents that both contribute Pb and organic carbon.

Overall, the analysis indicates that potentially toxic elements in the study area appears to be influenced by industrial and traffic emissions, followed by inputs from coal combustion and agricultural activities, while contributions from domestic sewage and organic matter are relatively minor. These findings suggest that regional pollution is controlled by multiple interacting sources, providing a scientific basis for subsequent pollution management strategies.

## 4. Discussion

### 4.1. Spatiotemporal Heterogeneity and Formation Mechanisms

As shown in Figure 2, PTEs exhibit clear spatial and vertical heterogeneity across sampling sites [52]. Elements such as As, Pb, and Zn show relatively stable profiles, whereas Cu and Cr display decreasing or fluctuating trends with depth [53], suggesting the influence of both external inputs and sedimentary processes [54].

The vertical profiles further indicate that relatively uniform distributions reflect stable sedimentary conditions [55,56]. whereas fluctuating trends (e.g., at site CY-3) suggest redox-related redistribution processes [57]. In addition, the strong correlations among Cu, Zn, Pb, and Ni indicate potential co-enrichment patterns, while Hg exhibits a distinct behavior [58]. implying different controlling factors [59].

### 4.2. Practical Implications of Ecological Risk

The Potential Ecological Risk Index (RI) at all four sampling sites falls within the “slight ecological risk” category (RI < 150), indicating an overall low ecological hazard [60]. Similar findings have been reported by Li et al. in sediments from reservoirs in South China, where RI values were generally below 150, although localized high-risk areas were influenced by As [9]. In the present study, however, the variability and median risk of As are notably higher than those of other elements [61], suggesting that As warrants particular attention in regional risk assessments. This observation aligns with the results of Luo et al., who noted that As in karst environments is prone to release under redox fluctuations, increasing ecological sensitivity [62,63].

The overall low-risk status, coupled with elevated As-related hazards at specific sites, implies two management priorities. However, As shows relatively higher risk levels compared to other elements, indicating localized ecological concern. This pattern is consistent with previous findings and highlights the need for targeted attention to As in the study area [64], increasing environmental risk. Second, for As and its homologous element group (e.g., As and Hg associated with coal combustion or pesticide sources identified by PCA and PMF) [65], higher-resolution source tracing and site-specific control measures are necessary to prevent local exceedances or amplified effects during ecologically sensitive periods [66]. This perspective is consistent with the findings of Deng et al. in karst reservoirs, who also recommended precise management of local risks associated with As and Hg [67]. The comparison with sediment quality guidelines is consistent with the RI results, both indicating an overall low ecological risk with localized moderate concern for certain elements.

### 4.3. Source Apportionment of PTEs in Sediments

The integrated use of correlation analysis, PCA, and the PMF model provides a mechanistic understanding of how different PTEs (PTEs) accumulate in reservoir sediments and how their sources differ under mixed land-use conditions. Rather than interpreting metals merely as statistical clusters, the combined evidence highlights the interplay between geochemical behavior, watershed processes, and human activities. It should be noted that correlation analysis and PCA are exploratory tools that reveal relationships and grouping patterns among variables, but they do not directly suggest pollution sources. Therefore, source interpretations based on these methods should be considered as indicative rather than conclusive, and are further supported by PMF results in this study.

The correlation analysis shows that Cu, Zn, Pb, and Ni exhibit strong co-variation patterns [68]. Such metal–metal coupling has also been reported, and this coherence among Cu, Zn, and Pb was attributed to their common association with fine particles derived from mechanical wear and metallurgical activities [69,70], suggesting potential common sources or similar environmental behaviors [71].

The PCA results deepen this mechanistic interpretation by showing that Cu, Pb, Zn, and Cr load strongly on PC1 [72]. By contrast, As and Hg dominate PC2, reflecting their known association with coal combustion residues, pesticide applications, and long-range atmospheric transport [73]. The intermediate loading of Ni indicates mixed contributions, who emphasized that Ni in reservoir sediments often reflects both natural geogenic inputs and diffuse anthropogenic enrichment.

**Figure 7:**
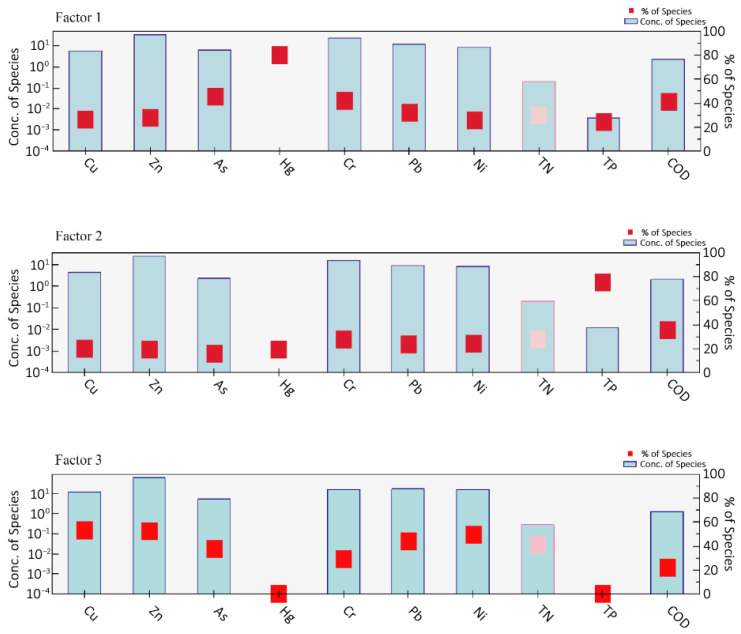
Heavy metal sources identified by PMF models and their contributions.

The PMF results (Figure 7) identify three major sources of PTEs. Factor 1 is characterized by high contributions of COD and TP suggesting inputs from domestic sewage and agricultural activities [74,75]. Factor 2 shows high loadings of Hg, As, and Cr, suggesting influences from coal combustion and agricultural inputs [18]. T Factor 3 is dominated by Cu, Zn, Ni, and Pb, indicating industrial and traffic-related sources. Overall, industrial and traffic-related sources may contribute significantly to PTE contamination in the study area [76].

Comparing these findings with previous studies in karst regions [77], industrial and traffic sources remain significant, but the relative importance of agricultural non-point inputs is higher in this tea-plantation-dominated catchment. This distinction reflects the unique land-use structure of tea-growing basins: steep slopes, high erosion sensitivity, and intensive agrochemical use can accelerate particulate-bound metal transport into reservoirs, amplifying the influence of agricultural activities relative to classical industrial sources.

These insights carry clear implications for watershed management. First, because agricultural runoff contributes substantially to As, Hg, and nutrient-associated metals, management should prioritize optimizing fertilizer and pesticide application schedules. Second, the PMF-derived association between TP and toxic metals underscores the need for structural measures—such as vegetated buffer strips, sedimentation ditches, or constructed wetlands—to intercept particle-bound pollutants along critical flow pathways. Third, the persistence of industrial- and traffic-related metals, despite limited local industry, highlights the importance of monitoring regional atmospheric deposition and strengthening soil conservation to minimize secondary remobilization. Collectively, these strategies reflect a shift from traditional point-source control to integrated watershed governance tailored to tea-plantation landscapes. It should be noted that the mechanistic interpretations proposed in this study (e.g., adsorption onto Fe/Mn oxides and organic complexation) are based on indirect evidence and literature support, as no direct measurements (such as Fe/Mn oxides or organic carbon fractions) were conducted. Therefore, these interpretations should be considered as tentative and require further verification in future studies.

## 5. Conclusions

The results of this study indicate that PTE concentrations exhibited clear spatial heterogeneity, with Zn (96.4–153 mg/kg) and Cr (43.2–64.6 mg/kg) showing relatively higher levels, while As ranged from 11.1 to 18 mg/kg. The pronounced co-enrichment of Cu, Zn, Pb, and Ni, together with the vertical differences among sampling sites, suggests that their spatial patterns are likely influenced by external inputs rather than natural background control. Multivariate analyses and the PMF model indicate that industrial and traffic-related sources are the main contributors, followed by agricultural inputs and coal combustion, while domestic sewage has a relatively smaller contribution. with domestic sewage and organic-rich runoff contributing to a lesser extent. The land-use characteristics of the tea-plantation watershed—especially fertilizer and pesticide applications—play a significant role in the accumulation of As and related elements. These quantitative results provide a clearer and more data-supported basis for understanding the distribution patterns and ecological risks of PTEs in the study area.

## Figures and Tables

**Table 1:** Statistical Results of PTEs Content (mg/kg).

Potentially Toxic Elements	Min (mg/kg)	Max (mg/kg)	Average Value (mg/kg)	SD (mg/kg)	Coefficient of Variation (%)	Background Value (mg/kg)	Excessive Multiple
Cu	19.8	25.4	21.99	2.78	12.64	32.00	-
Zn	96.4	153	123.43	22.52	18.24	99.50	1.24
As	11.1	18	14.66	3.19	21.76	20.00	-
Hg	0.27	1.97	1.12	0.56	50.7	0.11	10.2
Cr	43.2	64.6	55.51	7.18	12.93	95.90	-
Pb	31.3	45.3	39.69	4.37	11.01	35.20	1.12
Ni	29.6	36	33.08	3.50	10.58	39.10	-

**Table 2:** Potential ecological risk index.

$\mathrm{PTEs}\;E_{r}^{i}$								RI	Grade
Location	Cu	Zn	As	Hg	Cr	Pb	Ni		
CY-1	13.30	4.54	24.30	0.03	3.96	20.99	16.04	83.15	Minor ecological hazard
CY-2	14.00	4.96	28.40	0.03	4.41	22.87	16.89	91.55	Minor ecological hazard
CY-3	15.38	5.76	36.75	0.01	5.26	25.13	18.40	106.69	Minor ecological hazard
CY-4	12.31	4.60	27.80	0.08	4.90	21.22	16.34	87.25	Minor ecological hazard

## Data Availability

The raw data supporting the conclusions of this article will be made available by the authors upon request.
